# Current Progress and Challenges for Skeletal Muscle Differentiation from Human Pluripotent Stem Cells Using Transgene-Free Approaches

**DOI:** 10.1155/2018/6241681

**Published:** 2018-04-11

**Authors:** Nunnapas Jiwlawat, Eileen Lynch, Jeremy Jeffrey, Jonathan M. Van Dyke, Masatoshi Suzuki

**Affiliations:** ^1^Department of Comparative Biosciences, University of Wisconsin, Madison, WI, USA; ^2^The Stem Cell and Regenerative Medicine Center, University of Wisconsin, Madison, WI, USA

## Abstract

Neuromuscular diseases are caused by functional defects of skeletal muscles, directly via muscle pathology or indirectly via disruption of the nervous system. Extensive studies have been performed to improve the outcomes of therapies; however, effective treatment strategies have not been fully established for any major neuromuscular disease. Human pluripotent stem cells have a great capacity to differentiate into myogenic progenitors and skeletal myocytes for use in treating and modeling neuromuscular diseases. Recent advances have allowed the creation of patient-derived stem cells, which can be used as a unique platform for comprehensive study of disease mechanisms, *in vitro* drug screening, and potential new cell-based therapies. In the last decade, a number of methods have been developed to derive skeletal muscle cells from human pluripotent stem cells. By controlling the process of myogenesis using transcription factors and signaling molecules, human pluripotent stem cells can be directed to differentiate into cell types observed during muscle development. In this review, we highlight signaling pathways relevant to the formation of muscle tissue during embryonic development. We then summarize current methods to differentiate human pluripotent stem cells toward the myogenic lineage, specifically focusing on transgene-free approaches. Lastly, we discuss existing challenges for deriving skeletal myocytes and myogenic progenitors from human pluripotent stem cells.

## 1. Introduction

Recent advances in stem cell biology hold great promise for use in treating and modeling neuromuscular diseases [1]. Neuromuscular diseases affecting the function or development of skeletal muscle can arise directly via muscle pathology or indirectly via disruption of the nervous system. Despite devastating consequences, no effective treatment strategies exist in many cases, including muscular dystrophy. Attractive therapeutic strategies include the replacement of affected muscle cells with healthy myocytes or progenitor cells, thereby restoring skeletal muscle function.

Human pluripotent stem cells (PSCs), which include embryonic stem cells (ESCs) and induced pluripotent stem cells (iPSCs), represent a robust cell source for developing cell-based therapies targeting degenerating muscles as well as modeling neuromuscular disease conditions and for drug screening in culture. Particularly, iPSC technology allows creation of patient-derived stem cells, which can simulate pathophysiological conditions *in vitro* [2]. These *in vitro* models are expected to work as a unique platform for drug screening and allow comprehensive study of disease mechanisms.

In the last decade, a number of culture methods for myogenic differentiation from human PSCs have been published [3]. These include (1) transgene methods employing the direct manipulation of gene expression and (2) transgene-free methods employing pharmacologic inhibitors and agonists as well as isolated cytokines or other protein-based signals [3]. In this review, we discuss relevant pathways and events during skeletal muscle development which have been studied and manipulated in an effort to derive myogenic cell types from human PSCs. We then overview recent progress of the methods for myogenic derivation from human PSCs, specifically focusing on transgene-free approaches. Finally, we discuss the limitations and potential of these approaches for future treatment and modeling of neuromuscular diseases.

## 2. Skeletal Muscle Development and Molecular Networks

### 2.1. Embryonic Myogenesis and Terminal Differentiation into Myofibers

During early embryogenesis, the formation of skeletal muscle begins when the paraxial mesoderm segments form somites in response to signals from the notochord, neural tube, and surface ectoderm [4]. The developing somite then forms the dermomyotome, myotome, and sclerotome. The cells in the dermomyotome express the paired box transcription factors Pax3 and Pax7 [4–7]. The dorsomedial and ventrolateral portions of the dermomyotome give rise to the epaxial (primaxial) and hypaxial (abaxial) myotomes, respectively. Myf5-positive cells in the epaxial myotomes differentiate and form the trunk and back muscles. In contrast, MyoD-positive progenitors delaminate and migrate from the hypaxial myotome into the developing limb as the source of limb muscles. Myf5 and MyoD are expressed in committed muscle cells and are located in the myotome, which is formed from the maturation of dermomyotome lips [8–10].

**Figure 1:**
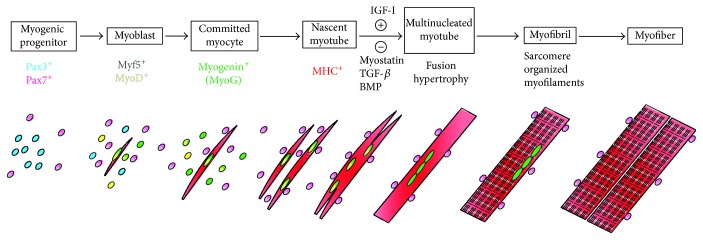
Skeletal muscle differentiation *in vitro.* The terminal differentiation starts when Pax3^+^ and/or Pax7^+^ progenitors begin to express Myf5 or MyoD as committed myoblasts. These myoblasts gradually express myogenin (MyoG) and form single-nucleated nascent myotubes with myosin heavy chain (MHC^+^). Insulin-like growth factor-I (IGF-I), TGF-*β*1 inhibitor, and myostatin inhibitors induce myotube fusion to form multinucleated myotubes. Actin, myosin, and elastic myofilaments are arranged to form organized sarcomeres within the myotubes. Organized sarcomere structures give rise to a striated pattern in the myotubes and represent the functional contraction unit of muscles.

**Figure 2:**
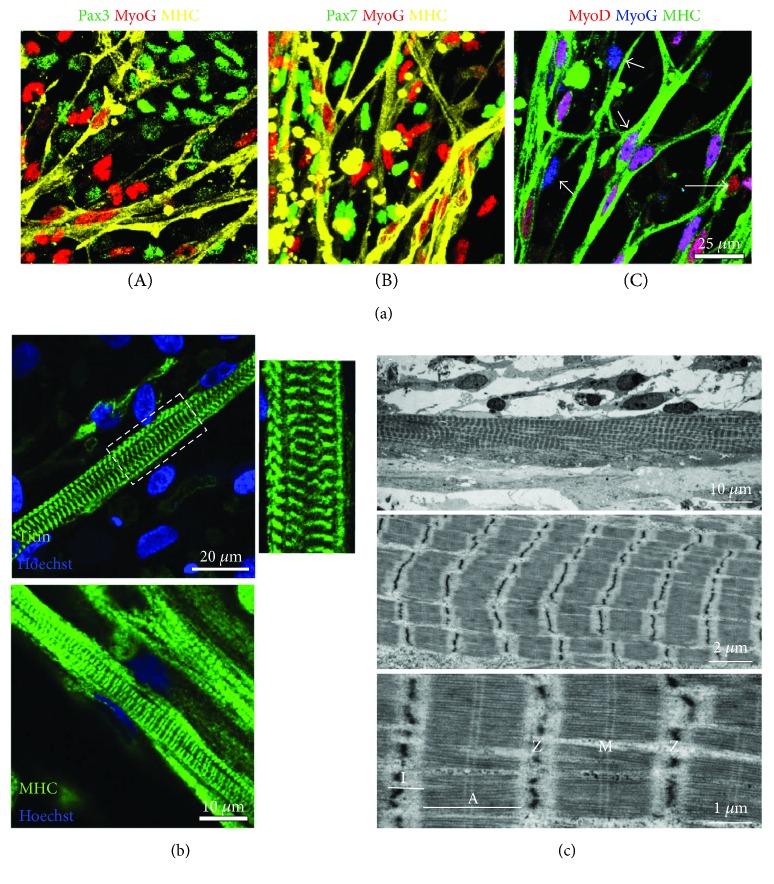
Derivation of skeletal myocytes and matured myotubes from human iPSCs using a transgene-free protocol. Human iPSCs can be sufficiently differentiated into myogenic progenitors and myotubes in a defined culture without genetic modification using free-floating spheres (EZ spheres) [59, 67]. (a) Human iPSC-derived myotubes were labeled with multiple myogenic proteins Pax3, Pax7, MyoD, myogenin (MyoG), and myosin heavy chain (MHC), demonstrating colocalization of those proteins in the same field. Some MyoD^+^ nuclei were overlapped with MyoG^+^ nuclei and fused on MHC^+^ myotubes (double arrow: MyoD^+^/MyoG^+^). The other nuclei were not overlapping with MHC but expressed either MyoD or MyoG (arrow: MyoD^+^/MyoG^−^; or arrow head: MyoD^−^/MyoG^+^) (C). Neither Pax3^+^ nuclei (A) nor Pax7^+^ nuclei (B) showed any localization with MyoG^+^ nuclei, which mostly fused on MHC^+^ myotubes. (b) Sarcomere formation in iPSC-derived myotubes. Titin staining revealed that striated patterns were clearly visible in the myotubes at 12 weeks MHC staining in the same cell preparations used for titin labeling. (c) Ultrastructures of iPSC-derived myotubes. After 12 weeks of terminal differentiation, mature sarcomeres were observed to be assembled into myofibrils. Morphological hallmarks, including I-band of actin filaments and A-band with distinct M line across myosin filaments, were clearly visible. Sarcomere Z lines appeared to be reasonably aligned and gave rise to a striated pattern. This figure is reproduced from Jiwlawat et al. [67] (under the Creative Commons Attribution license/public domain).

The terminal differentiation of progenitors and myoblasts initiates when myogenic progenitors in the dermomyotome stop dividing and exit the undifferentiated stage (Figure 1). Pax3- and/or Pax7-positive proliferating progenitors withdraw from the cell cycle once the differentiation step is initiated. These progenitors then become committed myoblasts expressing Myf5 and/or MyoD and form the nascent myotubes expressing myogenin and myosin heavy chain (MHC) (Figure 2(a)). Two waves of myotube formation occur during skeletal muscle development, sequentially giving rise to primary and secondary myotubes [4, 11]. Primary myotubes are generated from the fusion of early myoblasts and are aligned between muscle tendons to form the basis for embryonic muscle development. Late-stage myoblasts proliferate alongside primary myotubes and fuse to form secondary myotubes. As the secondary myotubes form, motor axons begin to innervate the embryonic muscle [11]. Single-nucleated myoblasts then fuse with the nearby myotubes to form multinucleated myotubes. Thick-myosin and thin-actin filaments within the myotube begin organizing and form sarcomeres, the functional units of muscle contraction. Sequential chains of sarcomeres, called myofibrils, align in maturing myotubes. Mature myotubes contain well-organized and aligned myofibrils which give rise to the characteristic striated pattern of skeletal myocytes (Figures 2(b) and 2(c)).

### 2.2. Signaling Molecules for Myogenesis

Myogenesis is delicately regulated by signaling events that influence proliferation and differentiation of stem cells and progenitor cells [4]. These events are driven by paracrine and/or autocrine signaling molecules that pattern and generate specific cellular lineages. A number of signaling molecules have been characterized to play critical roles for specification and differentiation from the somite to the myotomes [12, 13]. Signaling molecules can also contribute to terminal differentiation of myoblasts and myotube formation. These molecules regulate the expression of myogenic genes and proteins and influence the growth and fusion of MHC-positive myotubes. This section will introduce several signaling molecules critical for myogenesis; however, this is not an exhaustive list.

Wnt signaling plays a significant role in the development of myogenic progenitors in the somite and the formation of committed myoblasts in later stages of myogenesis. A diverse family of Wnt proteins is secreted from the neural tube and ectoderm. Wnt1 [12] and Wnt3a [14, 15] are produced in the dorsal neural tube, while Wnt7a is expressed in the dorsal ectoderm [12], and Wnt5a is localized in the dorsal ectoderm and limb mesenchyme [14]. Wnt ligands bind to Frizzled (Fzd) receptors and take action through a canonical (*β*-catenin) pathway or noncanonical pathways [16]. In mouse explant cultures, Wnt1 can enhance Myf5 expression and affects epaxial muscle formation. In contrast, Wnt7a promotes MyoD expression and influences hypaxial myogenesis [12, 17]. The initial expression of Pax3 and Myf5 was decreased in mice lacking both Wnt1 and Wnt3a [15]. A Wnt antagonist Frzb1 inhibits myogenesis in presomitic mesoderm, but not in mature somites. When Frzb1 was injected in a pregnant mouse, the process of myogenesis was disturbed by the reduction of Myf5 expression [18]. An inhibitor of Wnt/*β*-catenin signaling (IWR1-endo) inhibits myotube formation in murine myotube culture [19]. Additionally, an inhibition of glycogen synthase kinase 3*β* (GSK3*β*) can promote mesoderm differentiation via activating Wnt pathways [20–22].

Sonic hedgehog (Shh) is secreted from the notochord and floor plate of the neural tube [23] and regulates myogenic progenitor proliferation and differentiation [24]. In zebrafish, the number of Pax3- and Pax7-positive cells was significantly increased by a knockdown of the Shh gene [24]. Shh shows positive effects on muscle development by directing progenitor cells to Myf5-/MyoD-positive committed myocytes in the myotome by downregulating Pax3/Pax7 expression [25]. A reduced level of Myf5 expression was observed in Shh-null mice, resulting in a loss of distal limb structures [26]. Shh also enhances myogenic differentiation by increasing MyoD expression. An implantation experiment using Affi-Gel agarose beads soaked with 100 *μ*g/ml N-Shh in the lumen of the neural tube showed that Shh activates both MyoD and a sclerotomal marker, Pax1, in quail embryos [27]. Shh also promotes sclerotome formation while inhibiting dermatome formation [23].

Fibroblast growth factors (FGFs), including FGF2 (or basic FGF, bFGF), are critical factors for controlling proliferation and differentiation of myogenic progenitors and myoblasts during myogenesis. FGF2 is known to inhibit the differentiation of myogenic progenitors into myotubes [28, 29], implying that FGF2 could be used to maintain the progenitors at an immature stage. Interestingly, in murine myoblast C2C12 cells, inhibition of the mitogen-activated protein kinase (MAPK) pathway, which is downstream of FGF, increased the expression of MyoD, myogenin, and MHC and led to more myoblast fusion [29]. Both paracrine and autocrine effects of FGFs are proposed, as myocytes have been found to express both FGF ligands and FGF receptors. FGF ligands can bind to four FGF receptors (FGFR1–4) with varying levels of affinity. FGFR1–4 are transmembrane tyrosine kinase receptors capable of activating various downstream signaling cascades. FGFR1, 2, and 4 are expressed in immortalized myoblast cell lines such as mouse Sol 8 cells. Inhibitory effects of myocyte differentiation by FGF molecules were only observed when FGFR1 and 2 were presented in Sol 8 cells. Myogenic differentiation was stimulated when FGFR1 signals were inhibited by overexpressing truncated FGFR1 molecules [28]. Another study using chromatin immunoprecipitation-on-chip analyses demonstrated that FGFR4 is a direct downstream target of Pax3 in mouse embryo [30]. Further studies are necessary to elucidate which FGFRs are involved in modulating myogenesis. In addition, application of FGF2 or forskolin to C2C12 mouse myoblasts resulted in phosphorylation and activation of cyclic AMP response binding (CREB) protein. A gain-of-function mutation in CREB increased myoblast proliferation [31], indicating involvement of CREB signaling in myogenesis. Loss of CREB activity significantly decreased Pax3, Myf5, and MyoD expression in mouse embryos [17].

Both bone morphogenetic protein 4 (BMP4) and Notch enhance progenitor proliferation but inhibit muscle differentiation [25]. BMP4, secreted from the lateral plate mesoderm, sustains Pax3 expression and delays Myf5 and MyoD expression in chicken embryos [32]. An increased level of a BMP4 inhibitor Noggin in the dorsomedial lip of the dermomyotome of chick embryos inhibits BMP signaling and increases medial, rather than lateral, somite patterning [33]. Noggin-soaked bead implants promote muscle differentiation in chick embryos [34]. While BMP4 works as a secreted factor, an activation of Notch signaling requires direct cell-cell contacts. The Notch receptor is a single-pass transmembrane protein. Notch ligands bind to the extracellular domain of the receptor and then lead to proteolytic cleavage at the intracellular domain. After the intracellular domain is released, it migrates toward nucleases and modulates the expression of downstream genes [35]. A subset of migrating neural crest cells expresses a Notch ligand, Delta1. When chick embryo dermomyotomal cells transiently contact Delta1-expressing cells, expression of Myf5 and MyoD is activated. However, a prolonged contact with Delta1-expressing cells reverses the myogenic process resulting in Pax7-positive progenitor cells [36]. Notch signaling increases proliferation of myogenic progenitors but inhibits muscle differentiation by blocking MyoD transcriptional activity [37].

Transforming growth factor beta (TGF-*β*) and a TGF-*β* superfamily protein, myostatin, are known to modulate myogenic differentiation. TGF-*β* inhibits myogenic differentiation by suppressing the activity of myogenin [38]. However, a potent and selective inhibitor for TGF-*β* type I receptor (SB431542) and retinoic acid have been shown to rescue the negative effect of TGF-*β* on MHC^+^ myotube formation in C2C12 mouse myoblasts [39]. In mouse embryonic stem cells, a combination of TGF-*β* inhibitor (SB431542), a Wnt activator (BIO), and a Shh inhibitor (erismodegib) increased the expression of Pax7, Myf5, MyoD, and myogenin and the number of MHC^+^ myotubes [40]. Myostatin (also known as growth and differentiation factor-8, GDF-8) affects muscle cell differentiation in a manner similar to that of TGF-*β*. Dorsomorphin and LDN193189, which inhibit myostatin activity, significantly enhance myotube formation when applied to primary human myotubes and murine myotubes [41]. Follistatin, another myostatin inhibitor, increased fusion index and myogenic protein expression (including MyoD, Myf5, and myogenin) in C2C12 cells [42]. Another myostatin inhibitor, growth and differentiation factor-associated serum factor protein 1 (GASP-1), also enhances myogenin expression and fusion index in myotubes differentiated from C2C12 cells [43].

Insulin-like growth factor-I (IGF-I) is produced and secreted from myogenic cells and regulates muscle differentiation and growth. Both IGF-I receptors and IGF binding proteins are dramatically increased in mouse C2 myoblast cells during muscle differentiation [44]. IGF-I triggers terminal differentiation of myoblasts through the MAPK signaling pathway and increases protein expression of myogenin in murine C2C12 myotubes [29]. IGF-I, but not IGF-II, promotes myofiber fusion and hypertrophy in avian myotubes. This hypertrophy was promoted by increased synthesis and lower degradation of MHC proteins [45]. Interestingly, the steroid testosterone can stimulate fusion and hypertrophy of primary human myotubes via the IGF-I signaling pathway [46].

## 3. Derivation of Skeletal Muscle Cells from Human PSCs

Cell signaling plays a critical role in all stages of myogenesis. The timing of expression and the levels of signaling molecules are tightly controlled in order for the different stages of myogenesis to occur smoothly [12, 13]. Accumulated knowledge of the signaling pathways guiding myogenesis has aided the creation of a number of methods for deriving myogenic progenitors and myocytes from human PSCs. Current methods can be broadly categorized into two approaches: (1) induction of myogenic differentiation by overexpression of myogenic genes (transgene methods) and (2) derivation of myogenic progenitors under defined culture using growth factors and/or signaling molecules without transgenes (transgene-free methods).

### 3.1. Transgene-Based Approaches to Enhance Myogenic Differentiation

Selective induction of myogenic genes, such as the overexpression of PAX3, PAX7, and MYOD1, has been used in order to increase the efficiency of myogenic differentiation [3]. As discussed above, these transcription factors play critical roles in promoting proliferation and differentiation of myogenic progenitors and myoblasts during embryonic myogenesis. Different systems of gene expression, such as lentiviral and piggyback-based approaches, have been applied to transduce *PAX7* [47, 48] and *MYOD1* [49–52] genes into human PSCs. The transcription of myogenic genes can also be controlled by inducible gene expression systems such as tetracycline or tamoxifen [47–52]. These progenitors can be sufficiently enriched by fluorescence-activated cell sorting (FACS) if the transgene construct contains a fluorophore reporter gene like green fluorescent protein (GFP) and mCherry [47, 49].

One notable advantage of the transgene method is that transgene-based approaches can secure high efficiency of progenitor preparation (more than 90% in several methods). Typically, transgene methods yield progenitors more rapidly than transgene-free methods. However, as these approaches require an introduction of exogenous genes to the cells, the resulting cells may not fully reflect the normal processes of progenitor proliferation, differentiation, and maturation. Additionally, genetic modification remains a regulatory concern if the progenitors are to be used for cell-based therapy in patients. As such, myogenic progenitors prepared by transgene-free methods may be more suitable for transplantation in patients.

### 3.2. Transgene-Free Approaches: Myogenic Derivation under Defined Culture Conditions

Recent attempts have been made to derive myogenic progenitors from human iPSCs and ESCs under defined culture conditions using specific molecules secreted as paracrine factors that play important roles in muscle development (Table 1). These molecules control proliferation, migration, and differentiation from mesodermal cells into somite and dermomyotome [25]. FGF2 has been used at varying concentrations (5–100 ng/ml) to direct and enhance myogenic differentiation [20, 53–61]. Although 10–20 ng/ml FGF2 is commonly used to maintain proliferation in cell lines or primary cells, during our recent study, we found that a high concentration of FGF2 (100 ng/ml) significantly increased the number of Pax7-positive myogenic progenitors from human PSCs [59]. Other growth factors such as insulin-like growth factor-I (IGF-I), epidermal growth factor (EGF), hepatocyte growth factor (HGF), and platelet-derived growth factor (PDGF) have also been known to promote myogenic progenitor expansion and differentiation in human PSCs [57]. IGF-I can enhance myotube hyperplasia and fusion [62, 63]. IGF-I has been used at a concentration of 2–50 ng/ml to enhance terminal differentiation [55–57, 61, 64].

Small molecule inhibitors have also been used to direct and enhance myogenic differentiation. GSK3*β* inhibitors, such as CHIR99021 [55, 61] and BIO (6-bromoindirubin-3′-oxime) [20], can promote mesoderm induction during differentiation by activating Wnt pathways. CHIR99021 significantly enhances the expression of mesoderm genes such as *T*, *TBX6*, and *MSGN1* in human PSCs [54, 55, 65], indicating that this selective GSK3*β* inhibitor can promote mesoderm differentiation. While CHIR99021 has proven useful for *in vitro* mesoderm differentiation, it should be noted that it should only be used in culture for short periods and at a low concentration due to its toxicity [54, 55]. In fact, a longer exposure (more than 3 mM for 4 days) or a higher concentration (10 *μ*M for 2 days) of CHIR99021 results in toxicity in human PSC cultures [54, 55]. By contrast, a potent and reversible GSK3*β* inhibitor (BIO) demonstrates the lowest toxicity among other GSK3*β* inhibitors [20]. Further, an adenylyl cyclase activator, forskolin, has been used in a triple cocktail with FGF2 and a GSK3*β* inhibitor (BIO) to promote muscle differentiation [20].

Inhibitors of BMP type I receptors or TGF-*β* type I receptors, such as LDN193189 [56, 61, 64] and SB431542 [57], have been used to enhance derivation of a myogenic population from human PSCs. In some protocols, basal medium supplement of insulin-transferrin-selenium (commonly known as ITS) has been used to induce the initial step of mesodermal specification [53, 55, 66]. Oncostatin, necrosulfonamide, ascorbic acid, insulin, and dexamethasone were recently used in combination with growth factors and TGF-*β*1 inhibitors to increase skeletal myocyte derivation efficiency. These small molecules promoted a high percentage of skeletal muscle differentiation (up to 70% MHC^+^ myotubes) and shortened the differentiation period to less than a month [57]. A Notch antagonist DAPT (*γ*-secretase inhibitor) increased MyoD and myogenin gene expression [65]. A combination of CHIR99021 and DAPT synergistically enhanced myogenic differentiation [65]. Additionally, the rescue effect of LDN193189 and SB431542 mixture was demonstrated by the reduction of BMP4 levels and an increase of fusion index when applied to myotubes prepared from patient iPSCs with Duchenne muscular dystrophy [65].

## 4. Challenges for the Derivation of Skeletal Myocytes from Human PSCs Using Transgene-Free Methods

The evaluation of differentiation efficiency and myocyte maturity has been inconsistent between studies that focus on differentiating skeletal myocytes from stem cells. It would be of great benefit to the field to establish standards for these evaluations in order to more directly compare differentiation methods. Another challenge facing the field is that *in vitro* stem cell-derived skeletal myocytes often have an embryonic or perinatal phenotype. Additional bioengineering methods may be necessary in order to achieve skeletal muscle that is fully mature and therefore more physiologically relevant to *in vivo* skeletal muscle. In this section, we will discuss existing concerns of the current methods for preparing skeletal myocytes and myogenic progenitors from human PSCs, specifically related to transgene-free methods. However, several concerns are also applicable to transgene methods.

### 4.1. Differentiation Efficiency

Compared to when using transgene protocols, differentiation efficiency of skeletal myocytes overall still remains low when using transgene-free approaches. In order for the field to move forward toward goals of disease modeling, drug testing, and therapeutic development, differentiation efficiency should be improved. Currently, there is a wide range of reported efficiencies due to differences in reporting methods and the definitions used to describe the maturity of myogenic cell types. It is common to use stains for myogenic markers such as Pax7, MyoD, myogenin, and MHC. However, there is variation in how these stains are used to determine efficiency. Some protocols claim a very high efficiency rate of myogenic differentiation but often use a pooled percentage of Pax7, MyoD, myogenin, and/or MHC-positive cells. Others with lower efficiency may only be using one of the markers, which could be different from the marker chosen in another study. Along with the usage of immunocytochemistry for MHC, the counting of MHC^+^ cells in a field of view, the number of nuclei per myocyte, and the percentage of nuclei within myocytes (fusion index) have all been used to evaluate differentiation efficiency. Often, myocyte density and/or differentiation efficiency varies across a culture. Therefore, it is important to report the number of fields counted and how they were selected—specifically noting how bias was controlled. Overall, there is a need to standardize methods of calculating differentiation efficiency in order to facilitate comparisons between differentiation protocols.

### 4.2. Defining and Measuring the Extent of Myotube Maturation

In recent years, there have been a number of culture methods developed that yield MHC-positive skeletal myocytes from human pluripotent cells. Many of them require an extended culture period in comparison to methods for deriving other cell types. A method yielding myogenic progenitors or mature myocytes after a relatively short time would be of high significance to the field. However, it is also important to evaluate the maturity of cells yielded from rapid preparations. To date, it is difficult to compare the maturation state of myocytes generated by different methods due to differences in how each study defines maturity. Some focus on anatomical features, while others examine physiological functionality. Ideally, both aspects should be considered when evaluating myotube maturity. Studies taking an anatomical approach tend to use immunocytochemistry or electron microscopy to evaluate sarcomere formation and myofibril alignment as indicators of myotube maturity. Immunocytochemistry using antibodies against MHC or titin is a relatively accessible method to detect striations (Figure 2(b)); however, electron microscopy makes it possible to visualize sarcomeres at an ultrastructural level and examine sarcomeric organization and alignment (Figure 2(c)). It should be noted that in some preparations of maturing human PSC-derived myocytes, the results of immunocytochemical labeling of sarcomeric proteins (such as titin) may not correlate with ultrastructural results obtained through electron microscopy [67].

Some, but not all, studies take a physiological approach to determine myocyte maturation by examining the functionality of the cells. One method is to measure the frequency and coordination of spontaneous contractions observed in the differentiating myocytes. Contractions can be stimulated by a calcium flux or with addition of acetylcholine to the culture. Spontaneous contractions can also be observed shortly after culture medium changes [59, 67]. To properly examine spontaneous contractions, it should be taken into consideration when the cells were last given fresh media or were supplemented with the compounds that can promote contractions. Electrophysiology has also been used to monitor contractions and record contraction frequency and strength in cultured myotubes [48, 68]. Further, calcium imaging using dyes such as Fluo-3AM can be applied as an alternative method to electrophysiology. With a wide variety of methods currently being used, it is necessary to establish a preferred method of assessing physiological maturity of *in vitro* myocytes to better compare derivation methods. Another aspect of myocyte maturity is the fiber type expressed. During myogenesis, embryonic and slow type I MHC are expressed first. Then at later stages of maturation, myofibers develop glycolytic fast twitch MHC types IIa, IIb, and IIx [24, 69]. Commonly, MHC expression is examined using an antibody that reacts to all isoforms of MHC (such as MF20 clone), but a more detailed evaluation of MHC type would be useful for describing derived myocyte maturation.

In order to use human PSC-derived myocytes for *in vitro* modeling for adult-onset neuromuscular diseases, it is necessary to generate fully matured myotubes. However, iPSC-derived skeletal myocytes prepared using current methods typically are of an embryonic or perinatal phenotype. In addition to better understanding signaling molecules and the timing required for generating mature myocytes, bioengineering techniques will be needed to create surfaces recognized by human PSC-derived myocytes as appropriate for growth and maturation. Differentiation efficiency can likely be improved by controlling features such as surface coatings, adhesion ligands, and/or growth surfaces that encourage directionality and elongation. For instance, micropatterned surfaces can give myocytes much needed directionality [70]. It is likely that most two-dimensional culture environments are not similar enough to *in vivo* and that three-dimensional constructs will become necessary to encourage further stages of maturation [67]. Cocultures with motor neurons may support myotube maturation, as stimulation is required for proper contractility *in vivo* [71]. In the absence of motor neurons, myotubes can be chemically stimulated to contract by adding acetylcholine to the culture [72]. Electrical stimulation can also induce contractions and enhance maturation of myotubes [49, 73]. Further, mechanical stimulation may accelerate muscle differentiation and maturation [74].

### 4.3. Cell Enrichment and Large-Scale Expansion

While the way we report the efficiency of myogenic differentiation is valuable, it is also important to improve upon current methods to gain a pure population of myogenic progenitors and skeletal myocytes in culture. When prepared by a transgene-free method, the cultures commonly contain a heterogeneous cell population with myocytes and other cell types. Such heterogeneity influences the efficiency of *in vivo* engraftment following transplantation [75]. In order to improve differentiation efficiency, there is a need for more precise definition of which signal molecules to use and the timing of their use, but improved cell sorting techniques will also be necessary to further enrich derived myocytes. Fluorophore-labeled progenitors can easily be purified by FACS, if genetic modification is used [47, 49]. Also, several combinations of specific cell surface markers can be used to enrich myogenic progenitors and skeletal myocytes [55, 76–78]. Examples include combinations of CD54^+^/integrin *α*9*β*1^+^/SDC2^+^ [76], CD45^−^/CD11b^−^/GlyA^−^/CD31^−^/CD34^−^/CD56^int^/ITGA7^hi^ [77], CD56^+^/CD15^−^ [78], CXCR4^+^/C-MET^+^ [55], and HNK^−^/NCAM (CD56)^+^ [65, 79]. The most recent study indicated that a combination of two surface markers (ERBB3 and NGFR) can be applied to sufficiently purify a specific cellular population of human PSC-derived myogenic progenitors by FACS [79].

Another important consideration when developing derivation methods is whether they are adaptable to a large-scale expansion of myogenic progenitors and skeletal myocytes. Limited scalability seems to be a continued challenge among methods [75], which limits practical application and translation to patients as cell-based therapies. Often, cells are maintained in small quantities as a monolayer culture that is not always suitable for passaging. A recent study indicated that animal serum could promote cell expansion in PSC-derived myogenic progenitors, but the culture condition remained less defined [56]. However, a sphere-based culture may work to overcome this concern [59, 67]. As demonstrated in our recent study, human PSC-derived spherical cultures can be expanded for several weeks with specific signaling molecule supplementation in the medium [59, 67].

## 5. Conclusions

Valuable knowledge regarding the differentiation of myogenic progenitors and myotubes from human PSCs has been gradually accumulating [1, 3, 80–82]. Signaling molecules significantly contribute to generating a sufficient number of myogenic progenitors and myocytes from human ESCs and iPSCs without genetic modification. In addition to directing and enhancing differentiation of myogenic cells using signaling molecules, recent bioengineering approaches such as two-dimensional or three-dimensional culture, micropatterning, controlled stiffness, and mechanical, chemical, or electrical stimulation have enabled us to more accurately mimic the physiological environment of cultured cells while improving throughput, accuracy, and efficiency of *in vitro* analyses. A combination of signaling molecules and bioengineering approaches may further enhance the differentiation and maturation of human PSCs-derived myotubes for use in disease modeling, drug testing, and therapeutic development. Finally, *in vitro* cell models should represent similar morphological and physiological characteristics compared to tissues *in vivo*. In the skeletal muscle, fully mature myotubes have well-organized sarcomeres and the ability to contract in response to stimulation. In order to assess the maturity of human PSC-derived myotubes, it will be necessary to evaluate them using both anatomical and functional approaches.

## Figures and Tables

**Table 1:** Transgene-free methods of skeletal muscle differentiation using human pluripotent stem cells.

Reference	PSC type	PSC culture	Myogenic progenitor derivation and proliferation	Progenitor purification	Terminal differentiation	Efficiency of myogenic differentiation	*In vivo* engraftment	Use of disease-specific iPSCs
			Culture condition		Culture condition			
Barberi et al. 2007 [66]	ESCs	Feeder-dependent protocol using MEF	Monolayer cells were plated at 1 × 103 cells/cm^2^ on MEF, or 3 × 10^3^ cells/cm^2^ on feeder-freegelatin-coated plates, for 3-4 days under standard ESC culture conditions. The cells were then switched in DMEM/F12 supplemented with ITS for 20 days, and in *α*MEM, 10% FBS for an additional 14 days.	FACS based on CD73^+^/NCAM^+^. Sorted NCAM^+^ cells were grown in *α*MEM, 10% FBS until confluence.	The cells were differentiated in serum-free N2 medium.	60–80% of sorted NCAM^+^ cells were MyoD^+^. At 24 hours after exposure to N2 medium, approximately 7% and 46% of the total cells expressed Pax7^+^ and MyoG^+^, respectively. Upon terminal differentiation, MyoG, desmin, skeletal muscle actin, and myosin (MHC) were identified. Spontaneous twitching of myotubes was confirmed.	ESC-derived cells (5 × 10^5^ cells, CD73^+^/NCAM^+^ cells) were transplanted into a muscle injury model in SCID/Beige mice. The expression of reporter proteins (luciferase and GFP), human cell-specific nuclei, and laminin-positive myofibers were identified in the grafted muscles.	

Awaya et al. 2012 [53]	ESCs and iPSCs	Feeder-dependent protocol, using human ES cell maintenance medium (hESM)	EBs were formed by suspension in hESM for 7 days and then plated onto gelatin-coated tissue culture plates in ITS medium for an additional 14 days.		EBs were differentiated in skeletal muscle induction medium containing 10% FCS and 5% HS until day 112 of differentiation. In some experiments, dissociated EB cells (3 × 10^3^ cells/cm^2^) were seeded on collagen type I-coated plates. On day 49, the medium was changed to ITS medium.	xIn the cells migrating out of the EBs, the clusters Pax3^+^ and Pax7^+^ cells were randomly distributed at day 21. Skeletal myosin-positive multinucleated myofibers had appeared within most of the attached EBs at day 63.	Progenitors (1–5 × 10^5^ cells) were transplanted into the muscle of immunodeficient NOG (NOD/Shiscid/IL-2R*γ*null) mice following cardiotoxin injury. Four weeks after transplant, human cell-specific laminin A/C-positive nuclei were detected in the TA muscles. Further, the detection of human-specific laminin a2 proved that the transplanted cells produced human protein around the muscle fibers into which they had integrated.	

Xu et al. 2013 [20]	iPSCs	Feeder-free protocol using mTeSR on Matrigel-coated plates	EB culture in STEMdiff APEL Medium supplemented with 10 ng/ml FGF2, 0.5 *μ*M GSK3*β* inhibitor BIO, 20 *μ*M forskolin (“triple cocktail”) for 7 days.		EB cells were then cultured on Matrigel-coatedplates in DMEM, 2% HS for an additional 29 days.	Under terminal differentiation procedures (day 36), most of the cells expressed desmin (72%) and MyoG (92%), forming multinucleated myofibers. Sarcomere structures were also confirmed by electron microscopy.	iPSC-derived myogenic progenitors (1 × 10^5^ cells at day 14 of differentiation) were transplanted into cardiotoxin-injured muscles in NSG (NOD/SCID/IL-2R*γ*null) mice. Human *δ*-sarcoglycan expression in myofibers and colocalization of human-specific histone H2A and Pax7 were characterized in the grafted muscles.	

Borchin et al. 2013 [55]	ESCs and iPSCs	Feeder-free protocol using mTeSR1 on Matrigel-coated plates	PSC colonies were cultured in ITS medium (DMEM/F12 supplemented with ITS) in the presence of 3 *μ*M GSK3*β* inhibitor CHIR99021 for 4 days, and then in ITS medium containing 20 ng/ml FGF2 for an additional 14 days. The cells were then maintained in ITS medium alone for a further 17 days of culture in ITS medium alone.	FACS based on the expression of HNK, AChR, CXCR4, C-MET, following the differentiation for 35 days.	FACS-sorted AChR^+^ myocytes and CXCR4^−^/C-MET^+^ and CXCR4^+^/C-MET^+^ precursors were plated onto fibronectin/laminin-coated tissue culture wells in ITS medium supplemented with 10 *μ*M ROCK inhibitor Y-27632. The myocytes were maintained in ITS medium with 50 ng/ml IGF-I. The progenitor cells were differentiated in ITS medium.	In presorting cultures of CXCR4^−^/C-MET^+^ and CXCR4^+^/C-MET^+^ cells isolated at day 35 of differentiation, >18% Pax3^+^/Pax7^+^ and >8% MF20^+^ muscle cells were identified. In postsorting cultures at day 35, 97% in CXCR4^−^/C-MET^+^ and 98% in CXCR4^+^/C-MET^+^ were PAX3^+^; 84% in CXCR4^−^/C-MET^+^ and 96% in CXCR4^+^/C-MET^+^ were PAX7^+^. After 3 days of culture, few cells retained PAX7 expression, whereas all cells expressed MYH5. In postsorting cultures of AChR^+^ myocytes, all AChR^+^ cells were MyoG^+^ and MHC^+^ at 24 hours after plating.		

Hwang et al. 2013 [58]	ESCs (OCT4-GFP reporter line)	Feeder-dependent protocol using MEF	Single cells were cultured in suspension on ultra-low attachment plates to form EBs for 9 days, in high glucose DMEM containing 5% FBS, 2 mM l-glutamine, 100 nM dexamethasone, 100 *μ*M hydrocortisone, 1% penicillin/streptomycin, 1 mM transferrin, 86.1 *μ*M recombinant insulin, 2 mM progesterone, 10.01 mM putrescine, and 3.01 mM selenite. The EBs were then spilt, transferred, and cultured on a Matrigel-coated dish for an additional 8 days.	The cells growing out of the EBs were concentrated by FACS based on PDGFRA and OCT4-GFP.PDGFRA^+^ and PDGFRA^−^ cells were expanded in growth medium, containing high glucose DMEM, 10% FBS, 2 mM l-glutamine, and 1% penicillin/streptomycin.	PDGFRA^+^ and PDGFRA^−^ cells (1 × 10^4^ cells/cm^2^) were plated on gelatin-coated culture plates and differentiated in high glucose DMEM containing 2 mM l-glutamine, 100 nM dexamethasone, 100 mM hydrocortisone, 1% penicillin/streptomycin, 1 mM transferrin, 86.1 mM insulin, 2 mM progesterone, 10.01 mM putrescine, and 3.01 mM selenite with 10% FBS or without FBS.	The morphology of PDGFRA^+^ cells progressively became more spindle-like and fused and formed multinucleated myotubes (approximately 30% MHC^+^) after 14 days of terminal differentiation. In contrast, little or no myogenic differentiation was observed in PDGFRA^−^ cells population.	ESC-derived PDGFRA^+^ cells were transplanted into the muscle of NOD/SCID mice following cardiotoxin injury. Human laminin^+^ myofibers were identified after 14 days posttransplantation.	

Hosoyama et al. 2014 [59]	ESC and iPSCs	Feeder-dependent with MEF (ESCs) or feeder-independent protocols (iPSCs)	Sphere-based culture (EZ sphere) was maintained in Stemline medium containing 100 ng/ml FGF2, 100 ng/ml EGF, 5 ng/ml heparin sulfate for 6–12 weeks (42–84 days). The spheres were passaged by mechanical chopping every week.		Monolayer culture in high glucose DMEM, 2% B27 serum-free supplement on poly-l-lysine/laminin-coated coverslips.	Before terminal differentiation, progenitors were approximately 40% and 56% Pax7^+^, respectively. After 14 days of differentiation, the prevalence of Pax7^+^, MyoD^+^, 36–61% MyoG^+^, 24-25% MHC^+^ after 14 days of differentiation. Spontaneous contraction and AChR^+^ in myotubes were confirmed after 25 days of terminal differentiation.		The protocol was applied to patient-derivediPSCs (ALS with SOD1 or VAPB mutation, BMD, and SMA iPSC lines) for myogenic differentiation.

Shelton et al. 2014 [54] and 2016 [22]	ESCs	Feeder-free protocol in E8 medium	Monolayer cells (1.5 × 10^5^ cells per well) were plated on Matrigel-coated dishes in E8 medium supplemented with 10 *μ*M Y-27632 overnight. Cells were grown in E6 medium supplemented with 0.1% CHIR99021, BMP4, or activin-A for 2 days and then switched in unsupplemented E6 medium until day 12. From day 12 to 20, the medium was replaced with StemPro-34 media. Cells were then returned to E6 medium from day 20 to 35.		The cells were differentiated in N2 medium until the endpoint of the experiment.	Skeletal myocytes were prominent approximately 37% Pax7^+^ and 14% MHC^+^ by day 40 following 5 days of growth in N2 medium. Skeletal muscle contractions could be observed at this time point. When the cells were left in N2 media until day 50, 43% Pax7^+^ and 47% MHC^+^ were identified.		

Chal et al. 2015 [56] and 2016 [64]	ESCs and iPSCs	Feeder-free protocol in mTeSR1 media	Single cells from PSC colonies were seeded on Matrigel-coated plates (15,000–18,000 cells/cm^2^) in mTeSR medium supplemented with Y-27632 for 1 day. The medium was changed to a DMEM-based medium supplement with ITS, 3 *μ*M CHIR99021, and 0.5 *μ*M LDN193189 for 2 days. At day 3, 20 ng/ml FGF2 was added for an additional 3 days. At day 6, the cells were changed to a DMEM-based medium supplemented with 10 ng/ml HGF, 2 ng/ml IGF-I, 20 ng/ml FGF-2, and 0.5 mM LDN193189 for 2 days.		At day 8 in culture, the medium was changed to DMEM, 15% KSR, supplemented with 2 ng/ml IGF-I for 4 days and then supplemented with both 10 ng/ml HGF and 2 ng/ml IGF-I after day 12.	After 20 days, the cultures contained large fields comprising MHC^+^ and MyoG^+^ fibers and PAX7^+^ cells. By 4 weeks, ~22% nuclei were MyoG^+^ and 23% of nuclei were Pax7^+^. The muscle fibers showed sarcomeres, as demonstrated by titin and fast MHC staining. These striated fibers exhibited spontaneous twitching. The diameter of the muscle fibers was ~3.5 *μ*m.		

Caron et al. 2016 [57]	ESCs and iPSCs	Feeder-free protocol in serum-free M2 medium	Monolayer culture at 2500 cells/cm^2^ on collagen-coated plates and maintained in skeletal muscle induction medium containing 5% HS, 3 *μ*M CHIR99021, 2 *μ*M Alk5 inhibitor, 10 ng/ml EGF, 10 *μ*g/ml insulin, 0.4 *μ*g/ml dexamethasone, and 200 *μ*M ascorbic acid for 10 days.		At day 10, cells were dissociated with trypsin and replated at 2500 cells/cm^2^ onto collagen-coated plates and maintained for 8 days in skeletal myoblast medium containing 5% HS, 10 mg/ml insulin, 10 ng/ml EGF, 20 ng/ml HGF, 10 ng/ml PDGF, 20 ng/ml FGF2, 20 mg/ml oncostatin, 10 ng/ml IGF-I, 2 *μ*M SB431542, and 200 *μ*M ascorbic acid. After 18 days of differentiation, cells were maintained in myotube medium, containing 10 *μ*g/ml insulin, 20 *μ*g/ml oncostatin, 50 mM necrosulfonamide, and 200 *μ*M ascorbic acid, for 7 days.	After 10 days in skeletal muscle induction medium, 80% Pax3^+^, 20% Pax7^+^, and 30–40% CD56^+^ cells were identified. At day 18 (after the second step of differentiation), 50–60% of the cells were MyoD1^+^ and 20% desmin^+^. At day 26 (after the third and final stage of the process), 50–80% of the cells formed elongated and multinucleated myotubes that stained positive for MyoG, MHC, dystrophin, and *α*-actinin.		FSHD1- or BMD-affected ESCs were differentiated into MHC^+^ myotubes expressing MyoG. Patient-derived iPSCs with FSHD1 were also tested.

Choi et al. 2016 [65]	ESCs and iPSCs	Feeder-dependent protocol using MEF	At day 0, nonadherent cells were plated on a gelatin-coated dish (at 1.5 × 10^5^ cells per well of a 24-well plate), in MEF-conditioned N2 media containing 10 ng/ml FGF2 and 10 *μ*M Y-27632. At day 1, N2 media with 3 *μ*M GSK3*β* inhibitor CHIR99021 was added. At day 4, N2 media with 10 mM *γ*-secretase inhibitor DAPT were added until day 12. The resulting cells (“CHIR99021-DAPT culture”) were maintained in defined N2 media until day 30.		To determine the presence of fusion component myoblasts, the dissociated cells from the CHIR99021-DAPT culture (days 25–30) were also replated.	At day 30 in the CHIR99021-DAPT culture, approximately 63% of cells were MHC^+^ and 61% were MyoG^+^. The culture resulted in differentiation of myoblasts into multinucleated and spontaneously contractile myotubes with sarcomere structures. When the cells from the CHIR99021-DAPT culture were replated, the attached and surviving cells were mono-nucleated at day 2 after replating and then formed multinucleated myotubes at day 10 after replating with typical striations and expression of 35% dystrophin^+^, 37% titin^+^, and 40% *α*-actinin^+^.	The dissociated CHIR99021-DAPT culture cells (1–3 × 10^6^ cells) were transplanted into the injured TA muscle of NRG mice. At 6 weeks after transplantation, human nuclei (human-specific lamin A/C^+^) and human-specific laminin^+^ myofibers were detected in the grafted muscles.	Disease-specific cellular characteristics were characterized in the myotubes from patient-derived iPSC lines (FSHD, ALS with C9orf72 repeats, and DMD).

Swartz et al. 2016 [60]	iPSCs	Feeder-free culture on vitronectin-coated plates in TeSR-E8.	When iPSC colonies were ~250–400 *μ*m in diameter (day −1), 1.5% DMSO in TeSR-E8 medium was added. On day 0, cells were cultured in chemically defined medium (CDM) supplemented with 20 ng/ml FGF2, 10 *μ*M LY294002, 10 ng/ml BMP4, 10 *μ*M CHIR99021 (FLyBC) for 36 hours. Cells were then cultured in CDM supplemented with 20 ng/ml FGF-2 and 10 *μ*M LY294002 (FLy) for an additional 5.5 days. On day 7, cells were cultured in MB-1 and 15% FBS for 6 days. On day 12, the cells were cultured in fusion medium (2% HS in DMEM).		After 10 days of fusion medium (22 days total from the start of differentiation), the cells were changed to N2 medium (DMEM/F12 supplemented with 1% N2 supplement and 1% ITS).	At day 5, <5% of the total cells were Pax3^+^ mesodermal progenitors. At day 36, up to 64% (median 44.8%) of nuclei were MyoG^+^. A mix of intermediate- and late-stage muscle cells as demonstrated by desmin^+^ and MHC^+^. After 63 total days in fusion medium, brief and spontaneous contractions in a small set of myotubes were observed. Seven to 10 days after the addition of N2 medium, robust spontaneous contractions throughout the cell cultures were observed. Titin^+^ striation was displayed.		

Xi et al. 2017 [61]	ESCs and iPSCs	Feeder-free culture on Matrigel-coated plates in mTeSR1 medium.	On day −1, single cells from PSC colonies (25,000 cells/cm^2^) and seeded on Matrigel-coated plates in mTeSR1 medium containing 10 *μ*M Y-27632. On day 0, cells were switched to basal differentiation medium (BDM; DMEM/F12, 1% ITS and 0.5% penicillin–streptomycin) supplemented with 3 *μ*M CHIR99021 for 2 days. On day 2, cells were switched to BDM supplemented with 200 nM LDN193189 and 10 *μ*M SB431542 for another 2 days. On day 4, culture medium was changed to BDM supplemented with 3 *μ*M CHIR99021 and 20 ng/ml FGF2 for 2 days. On day 6, medium was switched to a KSR/HGF/IGF-I-based differentiation medium (DMEM, 0.5% penicillin–streptomycin and 15% KSR, 10 ng/ml HGF, 2 ng/ml IGF-I) for 14–21 days.		At day 29, cell suspension was filtered through cell strainers to exclude cell aggregates. Filtered cells were resuspended in SkGM2 medium supplemented with 20 ng/ml FGF-2 and replated at 15,000–20,000 cells onto Matrigel-coated plates. Cells were cultured for 7–10 days until reaching >70% confluency, and then medium was switched to N2 medium (BDM containing 1% N2 supplement) for 5 days.	At day 2, ~80% cells were Pax3^+^. Expression of myogenic markers was gradually increased toward day 20. At day 27, large areas of MHC^+^ cells emerged throughout the culture, and the majority also expressed titin. A high proportion of Pax7^+^, MyoD^+^, and MyoG^+^ was also identified. At day 44, approximately 58% MHC^+^ myocytes and myotubes were identified, as well as cells outside MHC^+^ area (6.5% Pax7^+^/MyoD^−^, 9.1% Pax7^−^/MyoD^+^, and 4.9% Pax7^+^/MyoD^+^).		

Hicks et al. 2017 [79]	ESCs and iPSCs	Feeder-free culture on Matrigel-coated plates in mTeSR1 medium.	For direct differentiation from PSCs, two published protocols (Shelton et al. 2014 [54] and Chal et al. 2015 [56], listed in Table 1) were used. Myogenic progenitors from day 50 culture were dissociated and filtered through 100 mm meshes.	FACS based on HNK^−^/NCAM^+^ or ERBB3^+^/NGFR^+^ were performed. Sorted cells could be grown in SkBM-2 media.	Myogenic progenitors were induced to differentiate in N2 media for 7 days. In some experiments, TGF-*β* inhibitors (SB431542 or A83-01) were added in differentiation media.	HNK^−^/NCAM^+^ enrichment increased PAX7 and MYF5 expression by ~1.7-fold in comparison to unsorted SMPCs. When differentiated in culture, the number of MHC^+^ cells was increased in HNK^−^/NCAM^+^ cells compared to replated/unsorted cells. ERBB3^+^/NGFR^+^ progenitors were enriched for *PAX7* and *MYF5* by 20-fold in comparison to ERBB3^−^/NGFR^−^ cells. ERBB3^+^/NGFR^+^ progenitors could form homogenous myotubes following terminal differentiation. TGF-*β* inhibitors (SB431542 or A83-01) significantly facilitated myotube fusion and maturation, as demonstrated by MHC protein levels (MYH1 and MYH8) and sarcomere formation.	The enriched cells (1 × 106 cells per 5 *μ*l, injected 5–10 *μ*l of cells) were transplanted into the injured or irradiated TA muscle of mdx-NRG mice. At 30 days after transplantation, human-specific lamin A/C^+^, spectrin^+^, and dystrophin^+^ myofibers were detected in the grafted muscle. HNK^−^/NCAM^+^ sorted cells survived in the grafted muscle but did not improve engraftment in comparison to unsorted cells. ERBB3^+^/NGFR^+^ progenitors significantly increased the number of engrafted myofibers in comparison to NCAM^+^ sorted cells.	Disease-specific iPSCs from DMD patients were used in this study. The mutation in DMD-iPSC lines was corrected by CRISPR-Cas9 gene editing, which could restore dystrophin expression.

AChR: acetylcholine receptor; *α*MEM: alpha minimum essential medium; ALS: amyotrophic lateral sclerosis; BMD: Becker muscular dystrophy; CXCR4: C-X-C chemokine receptor 4; DMD: Duchenne muscular dystrophy; DMEM: Dulbecco's modified Eagle's medium; DMSO: dimethyl sulfoxide; EB: embryoid body; EGF: epidermal growth factor; ERBB3: receptor tyrosine-protein kinase erbB-3; SC: embryonic stem cell; FACS: fluorescence-activated cell sorting; FBS: fetal bovine serum; FCS: fetal calf serum; FGF-2: fibroblast growth factor 2; FSHD: facioscapulohumeral muscular dystrophy; FTD: frontotemporal dementia; GFP: green fluorescent protein; GSK3*β*: glycogen synthase kinase 3b; HNK: human natural killer; HS: horse serum; HGF: hepatocyte growth factor; ITS: insulin-transferrin-selenium; iPSC: induced pluripotent stem cell; KSR: knockout serum replacement; MEF: mouse embryonic fibroblasts; MHC: myosin heavy chain; MYH1, MYH5, or MYH8: myosin heavy chain type 1, 5, or 8; MyoG: myogenin; NCAM: neural cell adhesion molecule (or CD56); NGFR: nerve growth factor receptor; NOD: nonobese diabetic; PDGF: platelet-derived growth factor receptor; PDGFRA: platelet-derived growth factor receptor-*α*; PSC: pluripotent stem cell; SCID: severe combined immunodeficiency; SMA: spinal muscular atrophy; SOD1: superoxide dismutase 1; TA: tibialis anterior; TGF-*β*: transforming growth factor beta; VAPB: vesicle-associated membrane protein/synaptobrevin-associated membrane protein B.
